# Introducing *NAR Molecular Medicine*, a new journal in the *Nucleic Acids Research* family

**DOI:** 10.1093/narmme/ugad002

**Published:** 2024-01-23

**Authors:** Maria Spies

**Affiliations:** Department of Biochemistry and Molecular Biology, University of Iowa Carver College of Medicine, 51 Newton Road, Iowa City, IA 52242, USA

Nucleic acids play important roles in all aspects of human physiology, while pathogenic mutations, changes in nucleic acid processing, structure and epigenetic modifications underlie a wide range of pathologies. Specific features of viral RNA are recognized by our innate immune system, and RNA, DNA and RNA/DNA metabolic processes can be targeted to fight off viral, bacterial and eukaryotic pathogens. The COVID-19 pandemic brought RNA viruses with their complex, constantly evolving genomes, but also the immense potential of RNA-based vaccines and therapeutics into the world’s spotlight. Additionally, the interplay between nucleic acids, enzymes and molecular machines manifests in countless ways that are highly relevant to medicine.

During its half-century service to the research community, *Nucleic Acids Research* has become a home journal to impactful mechanistic research involving all aspects of nucleic acids, as well as proteins, enzymes and nucleoprotein complexes modulating DNA and RNA. The addition of the online Methods, Database and Web Server issues, and the more recent launches of the *NAR Cancer* and *NAR Genomics and Bioinformatics* journals reflect the growth and importance of the *NAR* family to our burgeoning field. Still, there is a need for a venue that will specifically connect fundamental mechanisms of nucleic acids and disease. To address this need, we are introducing a new member of the *NAR* family. Our new journal, *NAR Molecular Medicine*, will be a rapid peer-reviewed, open access publication from our non-profit publisher that will maintain the standard of excellence of the *NAR* family. We will publish cutting-edge original research, critical reviews and case reports at the intersection of nucleic acid biology, therapeutics and the molecular mechanisms of disease. Our scope includes all areas of clinical medicine, pathology, molecular and cellular biology, structure–activity investigations related to disease, computation and drug discovery.

We welcome manuscripts that address the fundamental molecular mechanisms nucleic acids play in aging, immunology, behavioral and psychiatric disorders, cardiovascular, respiratory, hepatic, renal and metabolic diseases, neuromuscular degeneration, regenerative and reproductive medicine, understanding and manipulating innate immunity, as well as using nucleic acids and nucleoprotein complexes as therapeutics, drug targets, vaccines and agents in gene therapy. We will occupy space adjacent to, but not overlapping with, *NAR Cancer*.

Diving headfirst into building this new journal, I thought I had a good handle on the molecular events in health and disease that depend on DNA and RNA. However, talking to other *NAR* editors and my colleagues across the world and forming the Editorial Board helped me to realize how much broader the nucleic acids’ involvement is in molecular medicine. This is a rapidly growing and important field, and my plan for the journal is to fully embrace its numerous aspects.

As a newly appointed Editor-in-Chief of this exciting new journal, I am tremendously excited for its future. Building on the familiar *NAR* brand, I will be honored to continue the best *NAR* traditions of publishing in open access format, decision making by active scientists, and striving for fair and speedy reviews, which means I will need your help in peer review! To accommodate the breadth of topics the journal will cover, I will draw on the expertise of the Editorial Board composed of recognized authorities in various aspects of nucleic acids and disease, and reflecting the geographic and demographic diversity of the journal’s readers and authors. I am also looking forward to working with Shannon Rudden (*NAR Molecular Medicine* Managing Editor) and Liz Holwell (publisher) to provide a great experience to the authors and reviewers and to grow the journal into a good home for your best papers at the intersection of nucleic acid biology, therapeutics and the molecular mechanisms of disease.

The inaugural issue of *NAR Molecular Medicine* will open in January 2024, and we are accepting submissions. Please help us make this journal a success!

